# Multiple Myeloma: Cast Nephropathy, VTE, and Neurologic Complications

**Published:** 2013-01-01

**Authors:** Ellen Sullivan, Lisa C. Smith, Angela M. Falco

**Affiliations:** From Celgene Corporation (all authors are clinical nurse consultants)

## Abstract

Multiple myeloma accounts for approximately 1% of neoplastic diseases and 13% of hematologic cancers. Complications often associated with MM include neurologic and hematologic issues, infections, renal insufficiency, and bone involvement. It is crucial for advanced practice professionals caring for myeloma patients to assess patients accurately, be keenly aware of possible associated complications, and be familiar with appropriate interventions to prevent further injury. This article will provide an overview of MM-related renal insufficiency, with a focus on cast nephropathy, venous thromboembolism, and neurologic complications along with various causes and treatment options; a future article will address additional complications associated with MM.

Multiple myeloma (MM), also known as myeloma or plasma cell myeloma, is a progressive hematologic disease (Blade & Rosinol, 2007). This plasma cell disorder is characterized by clonal proliferation of malignant plasma cells in the bone marrow microenvironment, monoclonal protein in the blood or urine, and associated organ dysfunction (Palumbo & Anderson, 2011). Multiple myeloma accounts for approximately 1% of neoplastic diseases and 13% of hematologic cancers (Palumbo & Anderson, 2011). According to the International Myeloma Working Group, symptomatic myeloma diagnostic criteria include greater than 10% plasma cells in the bone marrow and/or the presence of biopsy-proven plasmacytoma, the presence of monoclonal protein in serum and/or urine, and more than one of the myeloma-related organ dysfunctions represented by the acronym CRAB (hyperCalcemia, Renal insufficiency, Anemia, or Bone disease).

Complications often associated with MM include neurologic and hematologic issues, infections, renal insufficiency, and bone involvement. It is crucial for advanced practice professionals caring for myeloma patients to assess patients accurately, be keenly aware of possible associated complications, and be familiar with appropriate interventions to prevent further injury. This article will provide an overview of MM-related renal insufficiency, with a focus on cast nephropathy, venous thromboembolism, and neurologic complications along with various causes and treatment options. Bone involvement, infections, and other hematologic complications including anemia, hypercalcemia, and hyperviscosity will be discussed in a future article.

## Renal Insufficiency in Multiple Myeloma

Approximately 20% of patients with newly diagnosed multiple myeloma present with renal failure. Moreover, this end-organ damage is the second most common cause of death in patients with MM (Dimopoulos et al., 2010a). Elevated serum creatinine levels are found in up to one-half of patients, with 20% having a creatinine level greater than 2 g/dL (Tariman, 2010). Multiple myeloma can affect the kidney through the filter, the tubules, or the tissue of the kidney itself. Other causes of renal impairment in patients with MM include acute tubulopathy, amyloid light-chain amyloidosis, light-chain deposition disease, tubulointerstitial nephritis associated with monotypic light-chain deposits, and plasma cell tumor nodules (Dimopoulos et al., 2010a). A biopsy-based diagnosis is important in the evaluation of patients with myeloma because each of the renal lesions has its own therapeutic and prognostic implications (Korbet & Schwartz, 2006). According to Tariman (2010), poor hydration and volume depletion; urinary tract infection and sepsis; nephrotoxins such as certain antibiotics, nonsteroidal anti-inflammatory drugs (NSAIDs), IV contrast dyes, and bisphosphonates; hypercalcemia and nephrocalcinosis; and hyperuricemia and urate nephropathy can lead to renal dysfunction.

## Cast Nephropathy

Cast nephropathy, the most common cause of myeloma-associated renal injury, occurs in at least 30% of patients (Dimopoulos et al., 2010a). This disorder, consisting of light-chain tubular damage, is often referred to as "myeloma kidney" and is the main cause of renal failure in patients with MM. Autopsy studies in patients with myeloma found cast nephropathy in 30% to 50% of cases (Korbet & Schwartz, 2006).

**Figure 1:**
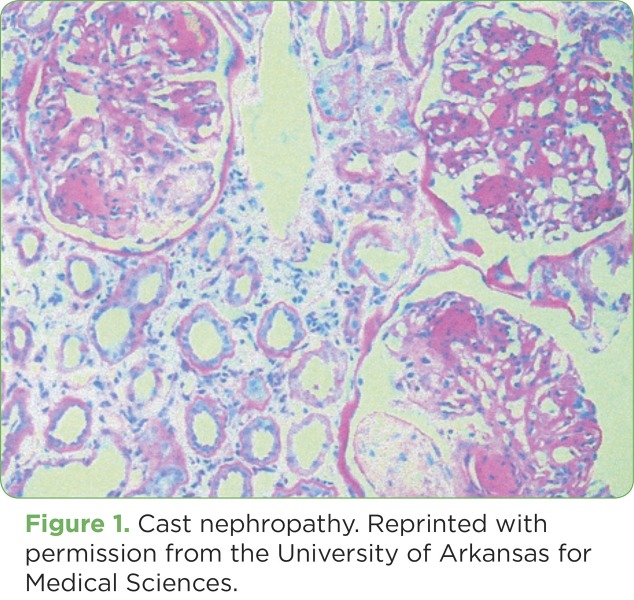
Figure 1. Cast nephropathy. Reprinted with permission from the University of Arkansas for Medical Sciences.

The major site of light-chain metabolism is the kidney, which has many filters called glomeruli. Blood passes through the glomeruli and enters the tubules, where light chains are filtered and catabolized by proximal tubular renal cells (Blade & Rosinol, 2007). This process is exceedingly efficient, and only a minute amount of light-chain protein normally appears in the urine. In the MM setting, abnormal proteins made by the plasma cell are present in the bloodstream and enter the tubules where they may encounter and connect with the Tamm-Horsfall protein, normally found in urine. Together, they form large casts, which lead to kidney damage by blocking the tubules inside the kidney (University of North Carolina [UNC] Kidney Center, 2012). Kidney biopsy is required for diagnosis of myeloma kidney (Figure 1).

Various factors such as dehydration, hypercalcemia, and use of NSAIDs can lead to a decreased glomerular filtration rate (GFR) and promote cast formation in myeloma patients. Dehydration can cause an increase in the plasma concentrations of light chains, which may exceed the proximal tubular cells’ capacity for absorption and catabolism. Hypercalcemia can reduce the GFR by inducing vasoconstriction, and drugs such as NSAIDs can reduce renal blood flow (UNC Kidney Center, 2012). There is a strong correlation between the degree of cast formation and the severity of renal failure. One important issue is that when a light chain is nephrotoxic, it usually causes renal failure from the beginning, even before other clinical manifestations of myeloma (Cibeirab, Blade, & Ludwig, 2009).

The reversibility of renal failure in patients with MM is highly variable (20%–60%). About 50% of patients with serum creatinine lower than 4 mg/dL recover a normal renal function. In contrast, for patients with a serum creatinine higher than 4 mg/dL the recovery rate is lower than 10% (Blade & Rosinol, 2007). In the past few years, the prognosis of these patients has improved due to more effective treatment of the disease and better supportive measures. In any event, the prognosis is strongly linked to the reversibility of renal function (Cibeirab, Blade, & Ludwig, 2009). Factors associated with renal recovery include serum creatinine < 4 mg/dL, 24-hour urine protein < 2 g/24 hr, and serum calcium > 11.5 mg/dL (Blade & Rosinol, 2007).

Clonal serum free light chain (FLC) levels can be measured with high sensitivity and specificity and used to rapidly screen for cast nephropathy. A sustained decrease in serum FLC levels within 3 weeks of starting treatment is associated with renal recovery; novel chemotherapy agents can maximize this early response (Cockwell & Hutchison, 2010).

Treatment for kidney disease associated with MM depends upon treating the myeloma itself. Approaches include alkylating-based conventional chemotherapy, high-dose therapy with autologous stem cell transplantation (Blade & Rosinol, 2007), bortezomib (Velcade) with high-dose dexamethasone, thalidomide (Thalomid), and lenalidomide (Revlimid). The role of plasma exchange in patients with suspected light-chain cast nephropathy and renal impairment is controversial (Dimopoulos et al., 2010b). See Table 1 for key factors to consider in the treatment of biopsy-confirmed cast nephropathy.

**Table 1:**
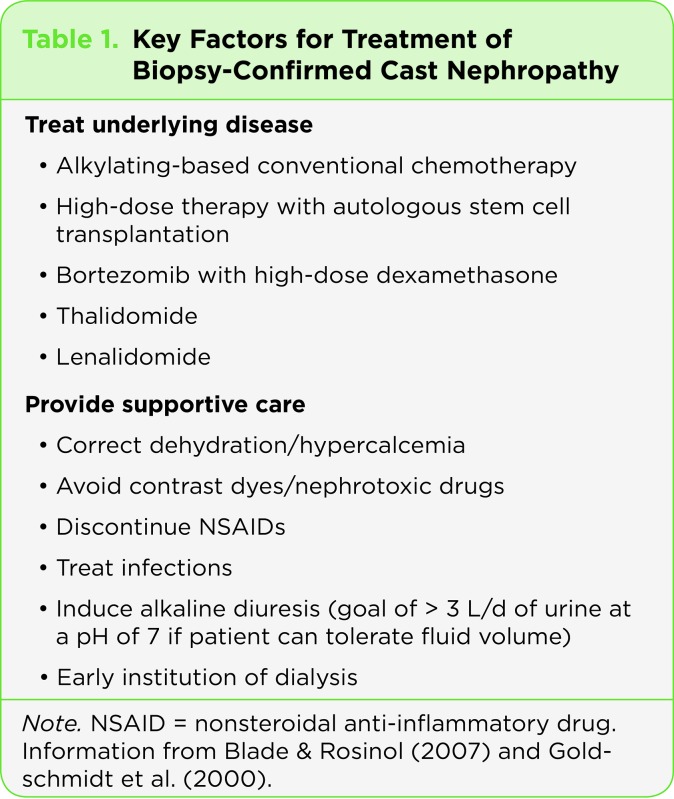
Table 1. Key Factors for Treatment of Biopsy-Confirmed Cast Nephropathy

## Neurologic Complications

The more common neurologic complications seen in patients with MM are spinal cord compression, nerve root compression, intracranial plasmacytomas, peripheral neuropathy, and leptomeningeal involvement (Blade & Rosinol, 2007). It is critical for advanced practice professionals to recognize the sequelae of these complications and administer appropriate treatment as needed.

## SPINAL CORD COMPRESSION

The most common and serious neurologic complication associated with MM is spinal cord compression due to a plasmacytoma arising from a vertebral body. Its incidence has been reported as 10%–20% (Blade & Rosinol, 2007). The thoracic spine is the most common site, followed by the lumbar and sacral regions. Patients can present with back pain, increased sensitivity (irritability), and paraparesis (weakness or partial paralysis) that can evolve over days or weeks. The onset can also be abrupt, with severe paraparesis occurring within a few hours. When the lumbar spine is involved, cauda equina syndrome can occur, with symptoms of low back and radicular pain and leg weakness (Gawler, 2004).

Spinal cord compression is a medical emergency that requires an urgent MRI when it is suspected. Treatment with dexamethasone plus radiation therapy must be initiated immediately if diagnosis is confirmed (Posner, 1987). In rare cases, spinal cord compression can be caused by vertebral collapse or spinal instability. Treatment in these cases involves urgent surgical decompression and insertion of a bone graft prosthesis (Gawler, 2004).

## NERVE ROOT COMPRESSION

Multiple myeloma patients can also develop radicular pain without evidence of spinal cord compression, which is referred to as nerve root compression. In this case, symptoms include back pain with "radicular metameric radiation" (Blade & Rosinol, 2007) and radicular sensory involvement. This can be interpreted as radiating pain or symptoms along the distribution of a nerve root or corresponding nerve roots in a structural pattern. Treatment is similar to that for spinal cord compression: dexamethasone and radiation therapy to relieve symptoms while waiting for systemic therapy to work (Posner, 1987).

## INTRACRANIAL PLASMACYTOMAS

Although the skull is frequently involved in MM, it is very rare to see intracranial plasmacytomas. Myeloma involvement of the skull base can, however, extend into the orbits, causing orbital pain, exophthalmos, and diplopia (Woodruff & Ireton, 1982). Diplopia can occur from the direct effect of an orbital plasmacytoma or from ophthalmoplegia caused by cranial nerve involvement within the orbits (Blade & Rosinol, 2007). In the evaluation of orbital involvement, CT scans are the most useful, but they must carefully explore all regions so as not to miss any lesions (Gawler, 2004). Another rare occurrence in this category is MM skull expansion resulting in subdural plasmacytoma, direct leptomeningeal infiltration, or brain plasmacytoma (Woodruff & Ireton, 1982). Primary intracranial plasmacytomas are extremely rare and can be associated with intratumor bleeding (Henson & Urich, 1982). With direct or hematogenous leptomeningeal involvement, the advanced practice professional can see spastic paraparesis on MRI imaging, suggesting a parasagittal meningioma (Gawler, 2004).

## PERIPHERAL NEUROPATHY

Peripheral neuropathy (PN) includes injury, inflammation, or degeneration of the peripheral nerve fibers. As it can affect the sensory, motor, and autonomic nervous systems, the advanced practice professional needs to fully assess each one of these areas. The incidence of PN can be found in over one-third of patients with MM; this is much higher than the incidence in the general population, which usually ranges from 2.4% to 8% (Tariman et al., 2008).

In some cases of PN, the M (monoclonal) protein plays a definite pathogenic role, for example, the neuropathy associated with immunoglobulin M (IgM) anti–myelin-associated glycoprotein (anti-MAG) and in patients with Waldenström macroglobulinemia and IgM monoclonal gammopathy of undetermined significance (Gawler, 2004). According to Latov, Hays, and Sherman (1988), anti-MAG antibodies are found in 50% to 60% of patients with IgM peripheral neuropathies.

Characteristics of IgM-associated neuropathies include sensory impairment and a milder course. The main disabilities are caused by hand tremors and gait ataxia (Nobile-Orazio, Meucci, Baldini, DiTroia, Scarlato, 2000). Improvement in anti-MAG–associated neuropathy has been reported with plasma exchange, chlorambucil (Leukeran), fludarabine, and rituximab (Rituxan; Blade & Rosinol, 2007). As noted by Nobile-Orazio et al. (2000), in an outcome analysis of 25 patients with neuropathy and high anti-MAG IgM after a mean follow-up of 8.5 years, the majority of patients had a favorable prognosis, even after several years. Additionally, current immune therapies, though temporarily effective in half of the patients, are associated with considerable side effects that limit their prolonged use and efficacy. This would suggest that until more effective or safer therapies become available, they should most likely be reserved for patients impaired in their daily life or in a progressive phase of the disease. The effect of treatment on the long-term prognosis of the neuropathies remains unclear (Nobile-Orazio et al., 2000).

The incidence of PN in patients with IgG or IgA multiple myeloma is lower than in those with the IgM type (Blade & Rosinol, 2007). This PN resembles chronic inflammatory demyelinating PN and can be either demyelinating or axonal. Patients with IgG- or IgA-associated PN usually respond better than those with the IgM type (Gawler, 2004). A cause of PN in patients with monoclonal gammopathies may be axonal degeneration related to amyloid deposition. It has been reported that severe orthostatic hypotension may result from autonomic nervous system involvement by amyloids (Blade & Rosinol, 2007).

## LEPTOMENINGEAL INVOLVEMENT

Leptomeningeal involvement of MM refers to involvement of the central nervous system (CNS) with detection of plasma cells in the cerebrospinal fluid (CSF), which is very unusual (Blade & Rosinol, 2007). A series of 25 cases of leptomeningeal involvement with MM seen at the University of Arkansas has been reported by Fassas et al. (2004), as well as the features seen in 71 reported cases that were reviewed. See Table 2 for a summary of their findings.

**Table 2:**
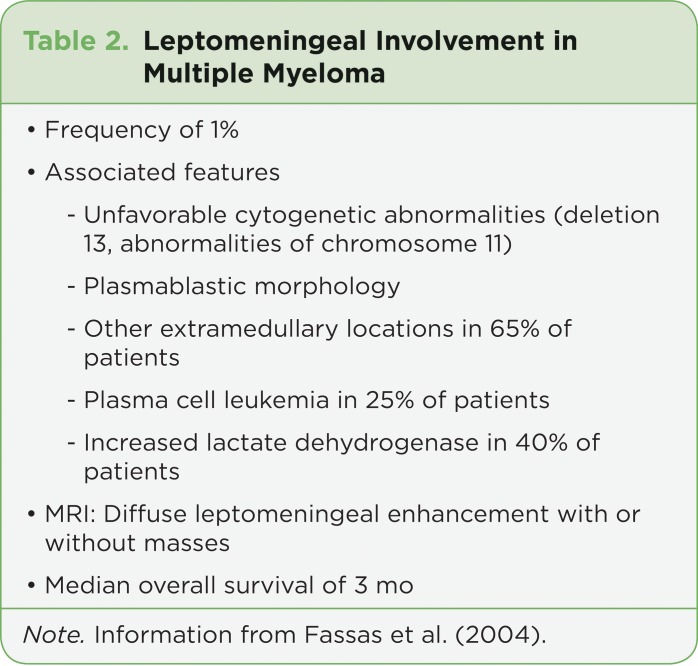
Table 2. Leptomeningeal Involvement in Multiple Myeloma

Although leptomeningeal involvement is very rare in MM (only about 1%), the advanced practice professional needs to be aware of the presenting symptoms. These include paraparesis, symptoms associated with increased intracranial pressure, cranial nerve palsies (particularly nerves V and IV), and confusion (Woodruff & Ireton, 1982). A CSF exam usually shows plasma cells with plasmablastic morphology, as well as increased protein levels and a positive immunofixation for the M protein (Blade & Rosinol, 2007). MRI results reveal a diffuse leptomeningeal enhancement, with or without additional findings such as a mass.

Treatment includes intrathecal therapy with methotrexate, hydrocortisone, cytarabine, cranial or cranial-spinal radiation, as well as systemic MM therapy (Fassas et al., 2004). Unfortunately. the prognosis is very poor, with a median survival of only 3 months from diagnosis of CNS involvement (Fassas et al., 2004).

## Venous Thromboembolism

Venous thromboembolism (VTE) is a well-known complication of MM that presents as a deep-vein thrombosis and/or pulmonary embolism. Along with other hematologic malignancies, MM carries the highest risk of VTE, with up to a 28-fold increase in risk compared to the general population (Blom, Doggen, Osanto, & Rosendaal, 2005). Although the development of VTE has not been shown to adversely affect overall survival or time to progression, this complication not only contributes to the morbidity of myeloma but also affects the patient’s quality of life and cost of care (Menon, Rajkumar, Lacy, Falco, & Palumbo, 2008; Zangari et al., 2010; Baz et al., 2005). Advanced practitioners caring for MM patients need to accurately assess each patient’s risk for VTE and provide appropriate thrombophylaxis measures to ideally prevent this complication.

While the exact mechanism of the pathogenesis of VTE in cancer still remains unclear, Falanga and Marchetti (2009) describe this process in the context of Virchow’s triad, which purports that clot formation is a process that involves abnormalities in three areas: blood flow (venous stasis), vessel wall injury, and a hypercoagulable state. Multiple myeloma causes numerous hemostatic abnormalities that will directly or indirectly affect all three areas of the triad, ultimately resulting in a prothrombotic state. Numerous manifestations of myeloma, such as lytic lesions, anemia, and fatigue associated with advanced or progressive disease, can have a negative impact on a patient’s mobility status contributing to increased venous stasis. Additionally, the presence of a paraprotein in the blood leads to a hyperviscous state, thereby reducing the velocity of blood flow. Central venous catheters and surgeries such as vertebroplasty and kyphoplasty lead to vascular wall injury and damage (Rome et al., 2008).

At the molecular level, endothelial injury is incurred via the proangiogenic microenvironment of myeloma, with increased production of vascular endothelial growth factor (Uaprasert, Voorhees, Mackman, & Key, 2010). Overexpression of adhesion molecules on both the tumor cells and vascular cells lead to increased interaction among the cells, adding to further vessel wall injury (Falanga & Marchetti, 2009; Hussein, 2006; Uaprasert et al., 2010). Multiple myeloma also greatly contributes to a hypercoagulable state via numerous mechanisms, including increased secretion of inflammatory cytokines (interleukin-6 and tumor necrosis factor–alpha) that upregulate the expression of tissue factor (a potent initiator of coagulation), paraprotein interference with fibrin structure resulting in delayed fibrinolysis, increased rates of acquired protein C resistance, and the presence of autoantibodies to inherent anticoagulants (Eby, 2009; Zangari et al., 2003; Menon et al., 2008; Uaprasert et al., 2010). Ultimately, all of these changes produce activation of the coagulation pathway coupled with a reduction in anticoagulation mechanisms, tipping the balance toward clot formation (Hussein, 2006).

## RISK FACTORS

Risk factors for VTE in MM are numerous and can be cumulative (Kristinsson, 2010; Niesvizky & Badros, 2010). In a review of over 1,700 MM patients from a managed care database, 66.3% of patients had at least 1 VTE risk factor and 48.3% had at least 2 risk factors (Brandenburg, Goss, Knight, Xiao, & Knight, 2008). Risk level can vary depending upon differences in individual factors. Table 3 presents VTE risk factors and recommendations for prophylaxis; Table 4 provides recommendations for treatment. It is also important to note that these risks can change over time (i.e., age, comorbidities, treatment regimens), highlighting the need for ongoing risk assessment with modifications to thromboprophylaxis as the risk level changes. Patient factors that increase risk include reduced mobility status, age, presence of a central line, and any recent hospitalization/surgery or acute illness such as infection. While routine screening for inherited thrombophilia is not currently recommended, a thorough history should be conducted to determine whether the patient or any family members have had clotting disorders that may suggest an inherited or genetic risk for VTE (Kristinsson, 2010; Palumbo et al., 2008). Comorbidities such as cardiovascular disease and diabetes, as well as certain medications, can also escalate the risk for thrombosis.

**Table 3:**
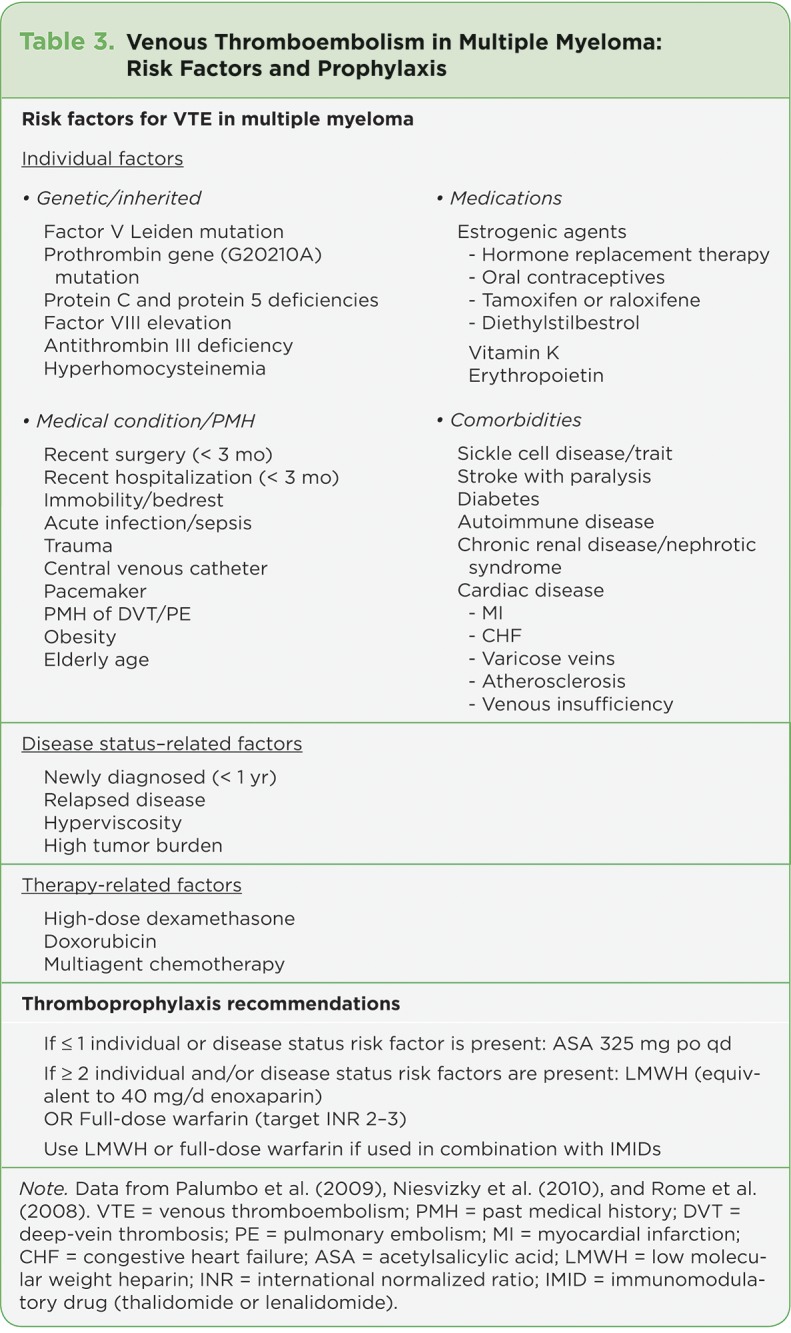
Table 3. Venous Thromboembolism in Multiple Myeloma: Risk Factors and Prophylaxis

Apart from a patient’s individual risk, clinical studies have identified both MM the disease as well as regimens used in its treatment as discrete risk factors for VTE (Palumbo et al., 2008; Uaprasert et al., 2010). Specific myeloma disease factors to consider are time from diagnosis, level of tumor burden, and the presence of hyperviscosity (Hussein 2006; Uaprasert et al., 2010, Kristinsson, 2010). In 2008, Kristinsson and colleagues identified 6,192 MM patients in a database of 4 million veterans. Compared with all other patients, the MM population had a 9.2-fold increase in deep-vein thrombosis risk, with the greatest risk observed during the first year following diagnosis (Kristinsson et al., 2008). Similarly, numerous clinical studies have documented VTEs occurring within the first few months of diagnosis, with a majority occurring within the first 2 to 6 months (Srkalovic et al., 2004; Palumbo et al., 2008).

Unusually high levels of procoagulant proteins, such as fibrinogen and factor VIII coagulant activity, have been documented in MM patients with advanced disease, supporting the theory that high tumor burden is also associated with increasing thrombosis risk (Auwerda, Sonneveld, de Maat, & Leeback, 2007; van Marion et al., 2008). Moreover, studies have documented higher rates of acquired resistance to activated protein C among MM patients, a condition that is associated with increased VTE risk. Patients who responded to treatment with a reduction in M-protein levels were documented to have decreasing levels of activated protein C resistance (Elice, Fink, Tricot, Barlogie & Zangari, 2006; Zangari et al., 2002).

More recently, the type of medication used to treat MM has been noted to dramatically affect VTE risk in MM patients. Prior to the introduction of the immunomodulatory (IMID) agents thalidomide and lenalidomide, the incidence of VTE in MM was lower than in many other malignancies (Rajkumar, 2005; Menon et al., 2008). A large part of this risk seems to be derived from the use of IMIDs in combination therapy vs. as single agents (Niesvizky & Badros, 2010; Palumbo et al., 2008). Rates of VTE with single-agent thalidomide use are approximately 5% in both newly diagnosed and relapsed refractory patients; however, when combined with dexamethasone, the risk increases 8-fold (El Accaoui, Shamseddeen & Taher, 2007). Studies examining the use of thalidomide as maintenance therapy have reported lower rates of VTE, probably due to low tumor burden (Uaprasert et al., 2010).

More importantly, the dose of dexamethasone is correlated to level of risk, with higher dosages being associated with increasing thrombotic complications. In a recent trial examining the use of lenalidomide in combination with low-dose dexamethasone (160 mg/month) vs. high-dose dexamethasone (480 mg/month), the incidence of VTE was 8% in the low-dose arm compared to 23% in the high-dose arm (Rajkumar et al., 2010). The use of IMIDs in combination with doxorubicin, erythropoietin-stimulating agents, and multiagent chemotherapy also increases the risk of VTE (Knight, DeLap, & Zledis, 2006; Kristinsson, 2010; Zangari et al., 2003; Palumbo et al., 2008).

Prior to initiating any treatment for myeloma, advanced practitioners need to assess baseline VTE risk incurred from the selected therapy along with any individual and disease-related risk factors. The overall goal of thromboprophylaxis should be to reduce the risk of VTE to less than 10% while using the safest and least cumbersome method for each individual (Palumbo et al., 2008). To date, ongoing clinical trials have demonstrated aspirin, low molecular weight heparin (LMWH), and full-dose warfarin as effective methods of reducing thrombotic complications (Pulmonary Embolism Prevention [PEP] Trial Group, 2000; Zonder et al., 2006; Palumbo et al., 2008; Kristinsson, 2010).

In contrast, the use of fixed low-dose warfarin has not been shown to be effective in VTE prevention (Zangari et al., 2004; Barlogie et al., 2006). Specific recommendations from the International Myeloma Working Group are outlined in Table 3.

It is recommended that all newly diagnosed patients who receive an IMID in combination with dexamethasone or chemotherapy be given some form of VTE prophylaxis (Palumbo et al., 2008). Aspirin may be considered in patients with only one individual or myeloma risk factor and for those using low-dose dexamethasone in combination with IMIDs. Full-dose warfarin or LMWH is indicated in patients with two or more risk factors or in those receiving IMIDs combined with high-dose dexamethasone, doxorubicin, or multiagent chemotherapy (Palumbo et al., 2008). The duration of prophylaxis will vary according to the length of treatment and change in disease status, with 4 to 6 months being cited as reasonable (Niesvizky & Badros, 2010; Kristinsson, 2010). Other factors to consider are a patient’s risk of severe thrombocytopenia, in which long-acting warfarin may be contraindicated, and avoidance of LMWH, which is renally excreted, in patients with renal failure (Paydas, 2008). In patients who experience VTE, the goals of treatment should be to identify the complication as quickly as possible, prevent embolization, and prevent recurrence of DVT (Niesvizky & Badros, 2010; Palumbo et al., 2008). Recommendations for the management of VTE in myeloma patients are also highlighted in Table 4.

## Implications for Advanced Practice

Disease-related complications can be numerous and may include renal insufficiency, anemia, bone marrow failure, hypercalcemia, bleeding disorders, infections, pathologic fractures, spinal cord compression, spinal cord and nerve root compression, intracranial plasmacytomas, and leptomeningeal involvement (Blade & Rosinol, 2007).

**Table 4:**
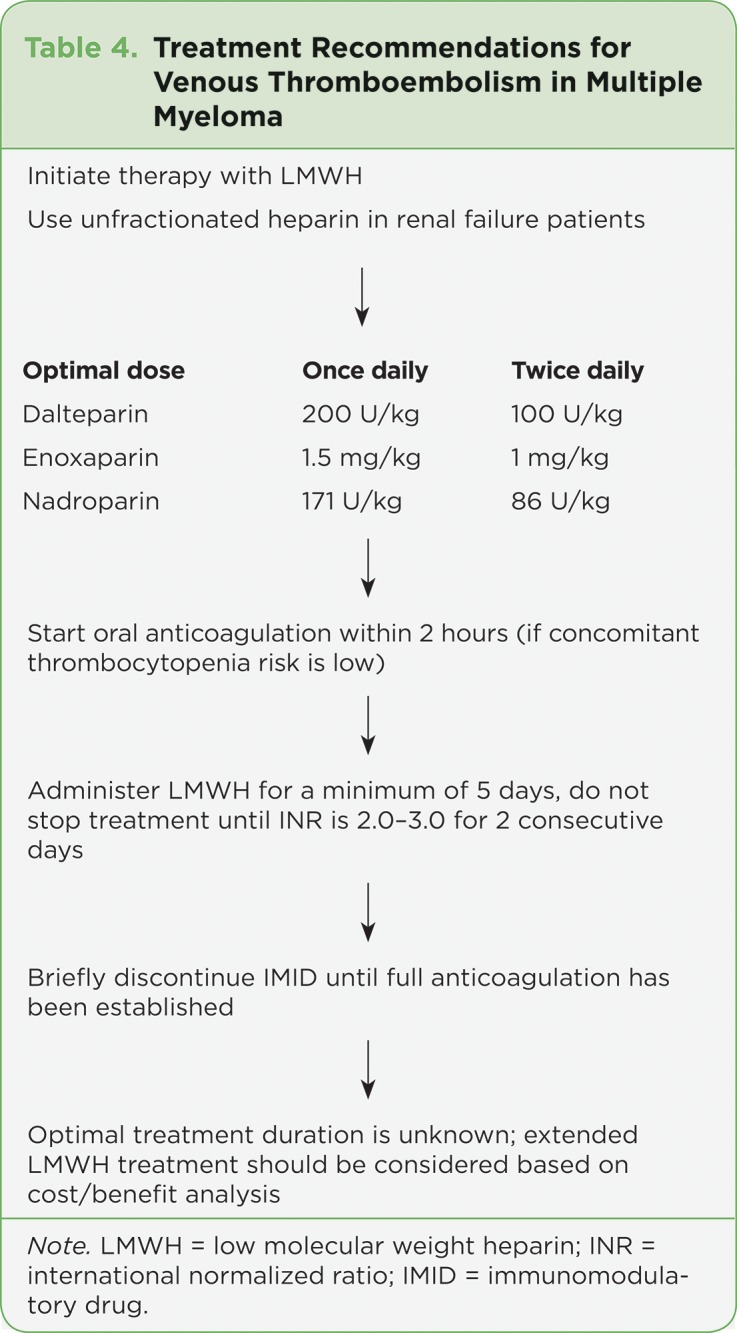
Table 4. Treatment Recommendations for Venous Thromboembolism in Multiple Myeloma

Identifying and providing early intervention is crucial to preventing further injury. Without expert assessment and appropriate interventions, complications associated with this complex progressive hematologic disease may become life threatening. Advanced practice professionals must have a thorough understanding of myeloma biology and the various complications to provide expert care to patients and improve overall quality of life.

## Conclusion

At any one time in the United States, there are approximately 100,000 people undergoing treatment for MM, with approximately 20,000 new cases being diagnosed each year (International Myeloma Foundation, 2012). In addition, according to Palumbo and Anderson (2011), the introduction of autologous stem-cell transplantation and the availability of agents such as thalidomide, lenalidomide, and bortezomib have changed the management of myeloma and extended overall survival.
